# Depression, antidepressant use, and breast cancer incidence in the Sister Study cohort

**DOI:** 10.1186/s13058-025-02043-4

**Published:** 2025-05-15

**Authors:** Mary V. Díaz-Santana, Jihye Park, Molly Rogers, Katie M. O’Brien, Hazel B. Nichols, Aimee A. D’Aloisio, Deborah B. Bookwalter, Dale P. Sandler, Clarice R. Weinberg

**Affiliations:** 1 Biostatistics and Computational Biology Branch, National Institute of Environmental Health Sciences, National Institutes of Health, 111 T.W. Alexander Dr., Durham, NC 27709 USA; 2 Epidemiology, Global Regulatory, Safety & Quality, GSK, Collegeville, PA USA; 3 Epidemiology Branch, National Institute of Environmental Health Sciences, National Institutes of Health, 111 T.W. Alexander Drive, Durham, NC USA 27709; 4 Department of Epidemiology, University of North Carolina Gillings School of Global Public Health, Chapel Hill, NC USA; 5 Social and Scientific Systems, Inc, 4505 Emperor Blvd, Suite 400, Durham, NC USA; 6 Westat, 1600 Research Blvd, Rockville, MD USA

**Keywords:** Breast Neoplasms, Depression, Depressive disorder, Antidepressant drugs, Antidepressive Agents, Breast, Selective Serotonin Reuptake Inhibitors, Overweight

## Abstract

**Background:**

Depression could affect breast cancer risk; however, epidemiologic findings are mixed. We assessed the association of breast cancer risk with self-reported history of diagnosed depression and time-dependent antidepressant use.

**Methods:**

We analyzed data from 45,746 women in the Sister Study cohort (2003–2009). Cox proportional hazard regression was used to estimate hazard ratios (HR) for breast cancer.

**Results:**

During follow-up (mean = 11.7 years), 3,899 breast cancers were diagnosed. There was no association between history of diagnosed depression and risk of breast cancer (HR = 0.98, 95%CI = 0.91–1.06). However, antidepressant use was associated with reduced risk of breast cancer (HR = 0.92, 95%CI = 0.85–1.00). Comparison of antidepressant drug classes revealed a suggestion of an inverse association with selective serotonin reuptake inhibitors (SSRIs, HR = 0.90, 95%CI = 0.81–1.00). Reduction was stronger in those with BMI < 25 (HR = 0.72, 95%CI = 0.59–0.89).

**Conclusions:**

Depression was not associated with breast cancer risk. We observed a suggestion of a reduction in risk associated with antidepressant use. The analysis evaluating the association by specific drug types, showed a suggestion of a reduction in breast cancer risk associated with use of SSRIs. The negative association with overall antidepressant use and SSRIs, was stronger in those with BMI < 25, which could reflect a dose effect. This was the first study to examine the association between depression, antidepressant use, and breast cancer risk in a large genetic-risk-enriched cohort.

**Supplementary Information:**

The online version contains supplementary material available at 10.1186/s13058-025-02043-4.

## Introduction

Depression is common in United States women, with an estimated age-standardized prevalence of 24% in 2020[1]. Antidepressants are the most frequently used therapeutic medication for depression with usage rates continuing to rise. Among U.S. women, antidepressant use has increased from 13.8% to 18.6% over the past decade. Depression has been hypothesized to increase breast cancer risk by influencing processes involved in cancer pathogenesis such as DNA repair, alterations in the immune system, and apoptosis.[2, 3] Furthermore, animal studies have suggested that antidepressant treatment, specifically selective serotonin reuptake inhibitors (SSRIs) and tricyclic antidepressants (TCAs), could increase breast cancer risk through their influence on elevated circulating prolactin levels. [4–11].

While there is thus biologic rationale linking depression to breast cancer risk, epidemiologic findings are not in agreement. Two prospective studies reported increased breast cancer risk among women with depression compared with healthy women.[12, 13] In contrast, two prospective studies that jointly evaluated associations with depression and antidepressant use reported no associations with either.[14, 15] More evidence is needed to clarify whether depression may increase breast cancer risk. If there is a positive association, mitigating this risk would be a public health priority given that breast cancer is common, and that 24% of U.S. women have depression.

Breast cancer risk associated with antidepressant use has been examined in multiple epidemiological studies, but findings are inconclusive. [16–33] A meta-analysis reported a pooled odds ratio of 1.11 (95%CI = 1.03–1.20), suggesting that antidepressants may be associated with a small increase in breast and ovarian cancer risk,[34] while more recent studies have not supported an association with breast cancer risk.[14, 15] Our study expands the current literature by examining this association in a large cohort of women with a family history of breast cancer.

Understanding whether antidepressant use affects breast cancer remains an important question because these medications are so commonly prescribed. Additionally, a variety of drugs are used to treat depression, and most studies have not considered them separately, allowing for possibly heterogeneous effects. One case–control study looked specifically at SSRI medications and found evidence for protection, which appeared driven by a stronger association in non-overweight (BMI < 25) women [30].

We here investigate the associations between depression, antidepressant use, and breast cancer risk using data from the Sister Study, a large nationwide prospective cohort of U.S. women with a first-degree family history of breast cancer. We additionally evaluate whether the associations vary by type of antidepressant or by body mass index (BMI) and explore possible effect measure modification by number of first-degree relatives with breast cancer.

## Materials and methods

### Study population

The Sister Study is a prospective cohort of women aged 35–74 years at enrollment, recruited across the U.S., including Puerto Rico, between 2003–2009.[35] Women were eligible if they had never been diagnosed with breast cancer but had at least one sister previously diagnosed. Baseline data collection consisted of in-home visits for collecting biospecimens and taking anthropometric measurements, comprehensive Computer Assisted Telephone Interviews (CATI), and self-completed questionnaires covering information on reproductive and medical history, and several lifestyle factors over the life course. Follow-up consisted of annual health updates on contact information and health status, as well as detailed follow-up questionnaires on changes in exposures and health status every 2–3 years.[35] The present research used data-release 10.1, which includes follow-up through October 2020. Response rates over time have been consistently high; more than 80% of participants had completed their most recent scheduled follow-up activity. All participants provided written informed consent. The Sister Study is overseen by the Institutional Review Board of the NIH.

We excluded women who were diagnosed with breast cancer before completing baseline activities (*n* = 59), had an unknown age at diagnosis (*n* = 17), withdrew their consent (n = 4), had uncertain breast cancer status (*n* = 5), or had zero follow-up time (*n* = 290). Women were further excluded if they were missing data on depression, antidepressant use, or potential confounders included in our multivariable model (*n* = 4,763), resulting in 45,746 women available for analysis.

### Measurement of depression

At baseline women were asked “Have you ever been diagnosed with clinical depression?” At follow-ups, updated information on a clinical depression diagnosis was collected, including age of diagnosis.

### Measurement of antidepressant use

Information on antidepressant use was obtained during the baseline interview and health follow-ups to capture information on current and past medications for specific conditions including depression.[36] They were also asked to report any additional medications not otherwise captured. Prior to their CATI, women were given booklets with the names of commonly used medications for specific medical conditions and told to use the booklet as a reference to identify drug names.[37] To minimize reporting errors, women were asked to have their current medications on hand during the interview.[37] Each reported medication was coded by product and class using the Slone Drug Dictionary (Boston University, Boston, MA, USA).[38].

At the first detailed follow-up, women were asked “do you currently take any prescription or non-prescription medications regularly or seasonally?”. Responses were open-ended and classified using the Slone Drug Dictionary. Medications were further classified by drug class SSRIs, TCAs, and serotonin and norepinephrine reuptake inhibitors (SNRIs)). Other classes of antidepressants were categorized as ‘miscellaneous’ anti-depressants. We also created an “other combinations” group for those who took antidepressant drugs from more than one class.

### Incident breast cancer

Women who reported incident breast cancer diagnosis during follow-up were asked for additional diagnosis information and permission to request medical records. We obtained medical records for 82% of incident breast cancer cases.[39] Agreement between self-reported breast cancer information and the medical records has been very high (99%).[39] Therefore, self-reported data were used, e.g. for invasive status, when medical records were not available.

### Statistical analysis

We used Cox regression to examine the association of breast cancer with clinical depression and antidepressant use, with age as the time scale. Self-report of ever-diagnosed depression and antidepressant usage was updated at the time of each available follow-up assessment, carrying values forward, as needed, when intermittent questionnaires were missing. In separate analyses, we examined the association of specific antidepressant drug classes and breast cancer. Women were followed from age at completion of intake questionnaires to age at breast cancer diagnosis, with censoring at death, loss to follow-up, or October 12, 2020. Because some participants had a sister who also enrolled, we used robust variance estimators to account for correlations between sisters due to shared genetic and environmental factors.

Potential confounders were identified using prior literature and selected using a directed acyclic graph.[40] All models were adjusted for the following confounders collected at baseline: race/ethnicity (non-Hispanic White/non-Hispanic Black/Hispanic/other), extent of family history of breast cancer (1 affected first-degree female relative, including half-sisters/≥ 2 affected first-degree female relatives), age at menarche (years, continuous), breastfeeding history (never to < 1 month/1 month to < 1 year/1- < 2 years/≥ 2 years), parity (nulliparous/1 birth/2 births/≥ 3 births), history of any hormonal contraceptive use (yes/no), history of hormone therapy use (never/unopposed estrogen/estrogen plus progestin), had oophorectomy (yes/no), age at first birth (nulliparous/< 20 years old/20 years old to < 30 years/30 years old to < 40/≥ 40 years old), physical activity level (metabolic equivalent of task [MET]-hours per week, continuous), smoking status (never smoked/past smoker/current smoker), alcohol intake (never drinker/former drinker/current drinker), had mammogram in prior 2 years (yes/no), alternate healthy eating index (AHEI) score[41] (continuous), highest level of attained education (high school diploma or less/some college or technical school/bachelor's or higher degree), and annual household income per person ($, continuous), with the exception of body mass index (BMI; kg/m^2^, continuous), which effect was updated with the information reported on the follow-up questionnaires. Further, we adjusted for baseline menopausal status (pre-menopause/post-menopause). Clinical depression models were also adjusted for the interaction between BMI and menopausal status. The reference group for the clinical depression models was women who reported having no history of being diagnosed with clinical depression. In the antidepressant use models, the reference group was non-users of antidepressants.

Outcomes considered were overall breast cancer (Invasive and ductal carcinoma in situ (DCIS)), with stratification by invasiveness, menopausal status, and estrogen receptor (ER) status. In the analyses of premenopausal breast cancer, women who transitioned from premenopause to postmenopause during follow-up were censored at menopause. Person-time occurring after menopause contributed to postmenopausal risk time.

A priori, we decided to assess possible effect-measure modification by BMI status (BMI < 25 kg/m^2^ vs. ≥ 25 kg/m^2^; updated for follow-ups), for the associations between clinical depression, antidepressant use, and breast cancer risk and we tested for statistical heterogeneity based on a Wald test of the anti-depressant-use-by-modifier interaction term. As an exploratory analysis, we also assessed effect measure modification by family history of breast cancer (one 1 st degree relative including half-sisters vs > 1) for the associations between clinical depression, antidepressant use and breast cancer risk. All statistical analyses were performed in SAS 9.4 (SAS Institute Inc.).

## Results

Among 45,746 women included in the analysis, at baseline, 9,410 women (20%) had ever been diagnosed with clinical depression *(*Table 1*)*. During an average of 11.7 years of follow-up, 3,899 reported a diagnosis of DCIS or invasive breast cancer.

**Table 1:** Characteristics of the Sister Study participants by Clinical Depression (*n* = 45,746)

	**History of Depression (*n* = 9,410)**	**No History of Depression (*n* = 36,336)**
**Characteristic**		
Age in years, mean (SD)	55.1 (8.3)	55.6 (9.1)
Follow-up time in years, mean (SD)	11.3 (3.3)	11.8 (3.2)
Race/Ethnicity, n (%)		
Non-Hispanic White	8104 (86.1%)	30,666 (84.4%)
Non-Hispanic Black	597 (6.3%)	3117 (8.6%)
Hispanic	442 (4.7%)	1617 (4.5%)
Other	267 (2.8%)	936 (2.6%)
Highest level of Education, n (%)		
High school/GED and lower	1304 (13.9%)	5564 (15.3%)
Associates degree/some college	3370 (35.8%)	11,934 (32.8%)
4-year degree or higher	4736 (50.3%)	18,838 (51.8%)
Annual household income per person in dollars, mean (SD)	40,052.7 (26,636.2)	43,144.9 (27,086.9)
First-degree family history of breast cancer, n (%)		
1 affected sister or half-sister	6877 (73.1%)	26,595 (73.2%)
2 + affected 1 st degree relatives	2533 (26.9%)	9741 (26.8%)
Age at menarche in years, mean (SD)	12.6 (1.5)	12.7 (1.5)
Parity, n (%)		
Nulliparous	1889 (20.1%)	6494 (17.9%)
1 birth	1511 (16.1%)	5130 (14.1%)
2 births	3435 (36.5%)	13,461 (37%)
3 + births	2575 (27.4%)	11,251 (31%)
Age at first birth, n(%)		
Never had a term pregnancy	1889 (20.1%)	6494 (17.9%)
< 20	1345 (14.3%)	4635 (12.8%)
20–29	4810 (51.1%)	19,838 (54.6%)
30–39	1297 (13.8%)	5101 (14%)
40 +	69 (0.7%)	268 (0.7%)
Breast feeding history, n (%)		
None/< 1 month	4548 (48.3%)	17,161 (47.2%)
1 month—< 1 year	2710 (28.8%)	10,436 (28.7%)
1- < 2 years	1204 (12.8%)	4937 (13.6%)
> = 2 years	948 (10.1%)	3802 (10.5%)
Menopausal status, n (%)		
Pre-menopausal	2913 (31%)	12,103 (33.3%)
Post-menopausal	6497 (69%)	24,233 (66.7%)
Ever used any hormonal contraceptive, n (%)	8363 (88.9%)	30,809 (84.8%)
Horomone Therapy Use, n (%)		
None	5020 (53.3%)	21,340 (58.7%)
Estrogen alone	2134 (22.7%)	6841 (18.8%)
Estrogen + Progestin	2256 (24%)	8155 (22.4%)
Had oophorectomy, n (%)	2631 (28%)	8415 (23.2%)
BMI (kg/m2), mean (SD)	28.9 (6.7)	27.1 (5.8)
Total physical activity in MET-hours/week, mean (SD)	11.3 (15.7)	15.2 (18.1)
Smoking status, n (%)		
Never Smoked	4759 (50.6%)	21,002 (57.8%)
Past Smoker	3567 (37.9%)	12,735 (35%)
Current Smoker	1084 (11.5%)	2599 (7.2%)
Alcohol drinking status, n (%)		
Never	259 (2.8%)	1415 (3.9%)
Former	1742 (18.5%)	5064 (13.9%)
Current	7409 (78.7%)	29,857 (82.2%)
Had mammogram in the past 2 years, n (%)	8937 (95%)	34,695 (95.5%)
AHEI score 2010, mean (SD)	59 (11.9)	60.3 (11.7)
Antidepressant Use, n (%)	6350 (67.5%)	3593 (9.9%)
Antidepressant Class, n (%)		
No AD	3073 (32.7%)	32,748 (90.1%)
SSRI alone	3304 (35.1%)	1860 (5.1%)
SNRI alone	873 (9.3%)	515 (1.4%)
TCA alone	222 (2.4%)	560 (1.5%)
Non-SSRI/SNRI/MAOI/TCA AD alone	837 (8.9%)	449 (1.2%)
Other AD combinations	1101 (11.7%)	204 (0.6%)

In multivariable analyses, there was very little association between history of clinical depression and breast cancer (HR = 0.98, 95%CI = 0.91–1.06). There was, however, an inverse association between time-dependent antidepressant use since baseline and both overall breast cancer risk (HR = 0.92, 95%CI = 0.85–1.00) and postmenopausal breast cancer risk (HR = 0.90, 95%CI = 0.83–0.99) (Table 2). Observed associations between history of clinical depression and breast cancer risk did not change after adjusting for use of antidepressants (SupplementalTable 1). When we assessed effect measure modification by BMI on the association between antidepressant use and breast cancer risk, we observed that in non-overweight women, antidepressants were associated with reduced HR for several outcome categories: overall (HR = 0.77, 95%CI = 0.66–0.89), invasive (HR = 0.78, 95%CI = 0.66–0.92), overall postmenopausal (HR = 0.74, 95%CI = 0.63–0.88), and invasive postmenopausal breast cancer (HR = 0.78, 95%CI = 0.64–0.94). These associations were not seen in women with overweight or obesity. Tests for heterogeneity by BMI were statistically significant (*p* < 0.05). Among non-overweight women, the antidepressant- breast cancer association was not dependent on ER status, but it was particularly strong for ER negative cancer: ER positive (HR = 0.83, 95%CI = 0.69–1.00) and ER negative (HR = 0.55, 95%CI = 0.32–0.95) (Table 3).

**Table 2:** HRs and 95%CIs for the associations between, clinical depression, antidepressant use, and breast cancer risk

	**Clinical Depression**		**Antidepressant Use**	
	**Exposed/Non-Exposed Cases**	**HR (95% CI)**	**Exposed/Non-Exposed Cases**	**HR (95% CI)**
Overall^a^	917/2982	0.98 (0.91, 1.06)	790/3109	0.92 (0.85, 1.00)
DCIS	190/630	0.99 (0.84, 1.17)	158/662	0.89 (0.74, 1.06)
Invasive	724/2347	0.98 (0.90, 1.07)	630/2441	0.93 (0.85, 1.02)
ER positive^b^	549/1743	1.00 (0.91, 1.10)	490/1802	0.98 (0.88, 1.08)
ER negative^b^	81/293	0.84 (0.66, 1.08)	69/305	0.78 (0.60, 1.03)
Overall Premenopausal	126/464	0.99 (0.81, 1.21)	118/472	1.04 (0.85, 1.28)
Overall Postmenopausal	791/2518	0.98 (0.90, 1.06)	672/2637	0.90 (0.83, 0.99)
Invasive Premenopausal	96/362	0.95 (0.76, 1.19)	89/369	0.99 (0.78, 1.26)
Invasive Postmenopausal	628/1985	0.98 (0.90, 1.08)	541/2072	0.92 (0.84, 1.02)

*Abbreviations* *DCIS* ductal carcinoma in situ, *ER* estrogen receptor

All models were adjusted for age, race/ethnicity, family history of breast cancer, age at menarche, breastfeeding history, parity, menopausal status, any hormonal contraceptive use, hormone therapy type, oophorectomy status, BMI, physical activity level, smoking status, alcohol intake, mammogram in prior 2 years, alternative healthy eating index score, education level, annual household income per person, age at first birth, and family history of breast cancer

Clinical Depression models (excluding by menopausal status) also adjusted for interaction between BMI and menopausal status at baseline

Reference group: For the clinical depression models (women without a clinical depression diagnosis); for the antidepressant use models (non-users of antidepressants)

^a^Includes Invasive and DCIS breast cancer

^b^*n* = 23 were excluded due to unknown ER status

**Table 3:** HRs and 95%CIs for the associations between, clinical depression, antidepressant use, and breast cancer risk; stratified by BMI category at Baseline

	**Clinical Depression**					**Antidepressant Use**				
	**BMI < 25 kg/m**^**2**^		**BMI ≥ 25 kg/m**^**2**^			**BMI < 25 kg/m**^**2**^		**BMI ≥ 25 kg/m**^**2**^		
	**Exposed/Non-Exposed Cases**	**HR (95% CI)**	**Exposed/Non-Exposed Cases**	**HR (95% CI)**	**Het**	**Exposed/Non-Exposed Cases**	**HR (95% CI)**	**Exposed/Non-Exposed Cases**	**HR (95% CI)**	**Het**
Overall^a^	272/1182	0.97 (0.85, 1.11)	645/1800	0.99 (0.91, 1.09)	0.87	213/1241	0.77 (0.66, 0.89)	577/1868	1.02 (0.92, 1.12)	< 0.001
DCIS	62/283	0.94 (0.71, 1.25)	128/347	1.02 (0.83, 1.25)	0.72	49/296	0.74 (0.54, 1.01)	109/366	0.99 (0.79, 1.23)	0.17
Invasive	208/898	0.98 (0.84, 1.14)	516/1449	0.99 (0.89, 1.09)	0.91	164/942	0.78 (0.66, 0.92)	466/1499	1.02 (0.92, 1.14)	0.01
ER positive^b^	164/675	1.02 (0.86, 1.21)	385/1068	1.00 (0.88, 1.12)	0.83	132/707	0.83 (0.69, 1.00)	358/1095	1.06 (0.94, 1.20)	0.03
ER negative^b^	21/112	0.77 (0.47, 1.24)	60/181	0.88 (0.65, 1.18)	0.64	15/118	0.55 (0.32, 0.96)	54/187	0.90 (0.66, 1.23)	0.13
Overall Premenopausal	49/247	0.91 (0.66, 1.24)	77/217	1.02 (0.79, 1.33)	0.60	44/252	0.88 (0.63, 1.22)	74/220	1.13 (0.86, 1.49)	0.26
Overall Postmenopausal	223/935	0.99 (0.86, 1.15)	568/1583	0.99 (0.90, 1.09)	0.92	169/989	0.74 (0.63, 0.88)	503/1648	1.00 (0.90, 1.11)	< 0.001
Invasive Premenopausal	33/189	0.78 (0.54, 1.14)	63/173	1.06 (0.79, 1.42)	0.22	31/191	0.80 (0.54, 1.17)	58/178	1.11 (0.82, 1.52)	0.20
Invasive Postmenopausal	175/709	1.03 (0.87, 1.21)	453/1276	0.98 (0.87, 1.09)	0.63	133/751	0.78 (0.64, 0.94)	408/1321	1.01 (0.90, 1.13)	0.02

*Abbreviations:* *DCIS* ductal carcinoma in situ, *ER* estrogen receptor

All models were adjusted for age, race/ethnicity, age at menarche, breastfeeding history, parity, menopausal status, any hormonal contraceptive use, hormone therapy type, oophorectomy status, physical activity level, smoking status, alcohol intake, mammogram in prior 2 years, alternative healthy eating index score, education level, annual household income per person, age at first birth, and family history of breast cancer

Reference group: For the clinical depression models (women without a clinical depression diagnosis); for the antidepressant use models (non-users of antidepressants)

^a^Includes Invasive and DCIS breast cancer

^b^*n* = 23 were excluded due to unknown ER status

To evaluate whether the associations with breast cancer risk varied by the extent of family history of breast cancer, we stratified our analyses by extent of family history. (Table 4). In these analyses we observed that, among women with more than one first-degree relative with breast cancer, there was an inverse association between antidepressant use and overall breast cancer (HR = 0.86, 95%CI = 0.75–0.98), invasive breast cancer (HR = 0.85, 95%CI = 0.73–0.99), and overall postmenopausal breast cancer (HR = 0.84, 95%CI = 0.73–0.97). Associations were consistent with 1.0 for women with only one first-degree relative with breast cancer. There were no associations between diagnosed depression and breast cancer risk for either group.

**Table 4:** Associations between, clinical depression, antidepressant use, and breast cancer risk; stratified by family history

	**Clinical Depression**					**Antidepressant Use**				
	**1 1st deg Relative**		** > 1 1st deg Relative**			**1 1st deg Relative**		** > 1 1st deg Relative**		
	**Exposed/Non-Exposed Cases**	**HR (95% CI)**	**Exposed/Non-Exposed Cases**	**HR (95% CI)**	**Het**	**Exposed/Non-Exposed Cases**	**HR (95% CI)**	**Exposed/Non-Exposed Cases**	**HR (95% CI)**	**Het**
Overall^a^	597/1925	1.00 (0.91, 1.10)	320/1057	0.95 (0.83, 1.07)	0.50	515/2007	0.96 (0.87, 1.06)	275/1102	0.86 (0.75, 0.98)	0.22
DCIS	112/404	0.92 (0.74, 1.14)	78/226	1.11 (0.86, 1.45)	0.28	96/420	0.88 (0.70, 1.10)	62/242	0.90 (0.67, 1.19)	0.93
Invasive	483/1518	1.02 (0.92, 1.14)	241/829	0.90 (0.78, 1.04)	0.18	417/1584	0.98 (0.88, 1.10)	213/857	0.85 (0.73, 0.99)	0.18
ER positive^b^	364/1116	1.05 (0.93, 1.18)	185/627	0.91 (0.77, 1.07)	0.20	325/1155	1.04 (0.92, 1.18)	165/647	0.87 (0.73, 1.03)	0.13
ER negative^b^	54/196	0.86 (0.63, 1.17)	27/97	0.81 (0.53, 1.24)	0.86	47/203	0.84 (0.61, 1.17)	22/102	0.68 (0.42, 1.08)	0.50
Overall Premenopausal	83/322	0.97 (0.76, 1.24)	43/142	1.07 (0.75, 1.52)	0.68	81/324	1.09 (0.85, 1.39)	37/148	0.98 (0.67, 1.44)	0.64
Overall Postmenopausal	514/1603	1.01 (0.91, 1.12)	277/915	0.93 (0.81, 1.06)	0.38	434/1683	0.94 (0.84, 1.05)	238/954	0.84 (0.73, 0.97)	0.31
Invasive Premenopausal	65/253	0.95 (0.72, 1.24)	31/109	1.00 (0.66, 1.50)	0.82	64/254	1.08 (0.81, 1.42)	25/115	0.85 (0.53, 1.34)	0.38
Invasive Postmenopausal	418/1265	1.03 (0.92, 1.16)	210/720	0.89 (0.76, 1.03)	0.14	353/1330	0.96 (0.86, 1.09)	188/742	0.85 (0.72, 1.00)	0.31

*Abbreviations:* *DCIS* ductal carcinoma in situ, *ER* estrogen receptor

All models were adjusted for age, race/ethnicity, age at menarche, breastfeeding history, parity, menopausal status, any hormonal contraceptive use, hormone therapy type, oophorectomy status, BMI, physical activity level, smoking status, alcohol intake, mammogram in prior 2 years, alternative healthy eating index score, education level, annual household income per person, and age at first birth

Clinical Depression models (excluding by menopausal status) also adjusted for interaction between BMI and menopausal status at baseline

Reference group: For the clinical depression models (women without a clinical depression diagnosis); for the antidepressant use models (non-users of antidepressants)

^a^Includes Invasive and DCIS breast cancer

^b^*n* = 23 were excluded due to unknown ER status

**Fig. 1:**
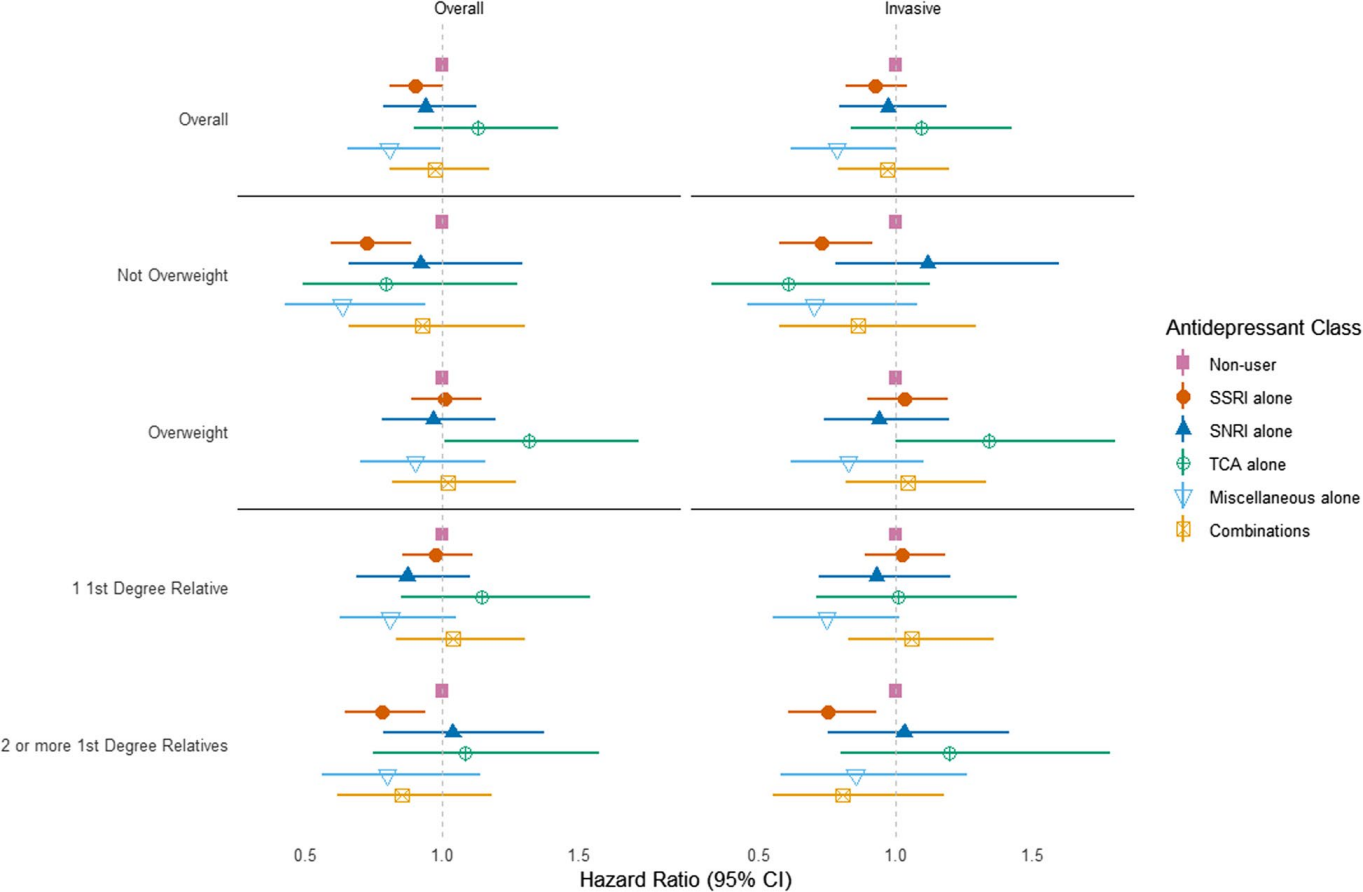
Associations between antidepressant classes and breast cancer risk

The associations between particular antidepressant medications, classified by drug class, and breast cancer risk are shown in Fig. 1 (See Supplemental Table 2 for individual HR and 95%CI). Compared to women who did not use antidepressants, we observed a reduction in overall breast cancer hazard associated with use of SSRIs alone (HR = 0.90, 95%CI = 0.81–1.00). Results from the stratified analyses by body mass index showed an inverse association with SSRIs among non-overweight women for both overall (HR = 0.72, 95%CI = 0.59–0.89) and invasive breast cancer (HR = 0.72, 95%CI = 0.57–0.91); the use of miscellaneous antidepressants alone in non-overweight women was also associated with reduced overall breast cancer hazard (HR = 0.63, 95%CI = 0.42–0.94). In overweight women, there was a positive association between use of TCA alone and both overall breast cancer (HR = 1.32, 95%CI = 1.01–1.73) and invasive breast cancer (HR = 1.34, 95%CI = 1.00–1.81). In analyses stratified by extent of family history, among women with more than one first-degree relative with breast cancer, a strong inverse association was observed between the use of SSRIs alone and both overall breast cancer (HR = 0.78, 95%CI = 0.65–0.94) and invasive breast cancer (HR = 0.75, 95%CI = 0.61–0.93), whereas HR’s were close to 1.0 for those with only one first-degree relative with breast cancer.

We also assessed whether the associations between AD use and breast cancer risk varied by the lifetime duration of antidepressant use. Most of the observed associations did not change with duration of use. However, the inverse associations with any anti-depressant use (HR = 0.83, 95%CI = 0.71–0.96; HR = 0.83, 95%CI = 0.70–0.99) (*Supplemental *Table 3) and with SSRIs (HR = 0.81, 95%CI = 0.67–0.97; HR = 0.76, 95%CI = 0.61–0.94) (Supplemental Table 4) among non-overweight women, for both overall and invasive breast cancer, were observed only among women who had been on anti-depressants (or specifically SSRI) for 4 or more years (Supplementary Table 3 & 4).

## Discussion

In this large prospective cohort study of women with a first-degree family history of breast cancer, clinical depression was not associated with breast cancer risk. However, antidepressant treatment was associated with reduction in risk, overall and for postmenopausal women. When considering specific drug types, SSRIs were associated with a reduction of overall breast cancer risk, particularly for non-overweight women and women with more than one affected first-degree relative. “Miscellaneous” antidepressants as a category was also inversely associated with breast cancer in non-overweight women, whereas TCA was associated with increased risk for overall and invasive breast cancer, but only in women with a BMI > 25 kg/m^2^.

In contrast to our observations, prior epidemiological studies have reported a positive association between depression and breast cancer risk. A meta-analysis using data from eight prospective studies assessing the relationship between depression and breast cancer risk, reported a statistically significant 59% increased risk of breast cancer among depressed women, compared to women without depression[42]. However, the studies included in that meta-analysis showed a wide variation in results, possibly due to incomplete adjustments for potential confounders, and differences in the definitions and assessment of depression and total follow-up time. Some of the inconsistencies in the results could be explained in part by confounding, many of the studies included in this meta-analysis lacked adjustment for important breast cancer risk factors such as mammogram use, alcohol use, and BMI. Two of the studies[43, 44] estimated a small inverse association with breast cancer risk. An association between depression and increased risk is not supported by our analysis. Our findings are more consistent with the results reported by two other large cohort studies[14, 15]: the Women’s Health Initiative[14] and the Nurses’ Health Study (NHS) and NHSII[15], both reported a null association between depression and breast cancer.

Previously reported results for the association between antidepressants and breast cancer are also inconsistent. Two prospective cohort studies[18, 33] reported an increased risk of breast cancer with anti-depressant use. One of these studies[33] used data from the Finland nationwide record linkage and reported a significant 53% increase in breast cancer risk associated with > 4 years of SSRI use. Anti-depressant use in that study was obtained from a nationwide prescription register. Similarly, the New York University Women’s Health Study estimated that the use of anti-depressants at baseline was associated with a 75% increased risk of breast cancer [18], but the number of cases in that category was small (*n* = 16). On the other hand, no association between anti-depressant use and breast cancer risk was found by several retrospective studies[22, 23, 29] and two prospective cohort studies[14, 15]. Both prospective cohort studies[14, 15], used self-reported data on antidepressant (AD) use, one from postmenopausal women in the Women’s Health Initiative[14] and the other [15] used data from women in the NHS and NHSII. The first had null findings for anti-depressants and the second was more consistent with ours, as they observed a non-statistically significant reduction in overall breast cancer, and postmenopausal breast cancer risk associated with anti-depressant use.

Our results suggest that antidepressant use is associated with a reduced risk of breast cancer, and that the association is particularly strong among thin women and women with a strong family history of breast cancer. The apparent effect measure modification by family history could explain the overall discrepancy between our findings and those of earlier cohort studies, since our cohort is restricted to women with a first-degree history of breast cancer: if SSRI somehow blocks effects of a causative genetic variant, the Sister Study, through its enriched sampling of women with familial risk, would have enhanced power to find it. However, we should interpret these results with caution, as they arose in an exploratory analysis.

Supporting that effect-blocking notion, a case control study[25] reported that first-degree family history of breast cancer modified the associations between antidepressant use and breast cancer risk (p-for interaction = 0.0017). They observed that among women with a first-degree family history of breast cancer, those on antidepressants had a 0.40 (OR = 0.40, 95%CI = 0.20–0.90) fold lower odds of breast cancer. However, those results were based on a small number of breast cancer cases (*n* = 15). Results from a meta-analysis showed that responses to antidepressants, particularly SSRIs, are different among individuals with the 5-HTTLPR polymorphism in the promoter region of the serotonin transporter (SLC6 A4)[45]. Although the mechanisms through which SSRIs would affect tumor development are unclear, SSRIs have been reported to inhibit tumor sphere formation in human breast cell lines, both *in v*itro and in vivo[46]. In addition, Fluoxetine (a type of SSRI) has been reported to induce apoptosis and autophagy-mediated cell death in several breast carcinoma cell lines[47, 48]. Future research is warranted to confirm and clarify possible inhibitory effects on carcinogenesis.

We also noted effect measure modification by BMI, with thinner women showing a stronger negative association with SSRI. This difference could possibly reflect a dose effect if there is a standard dose prescribed, because thinner women have lower blood volume and an effectively larger dose to the breasts. Alternatively, the apparent effect measure modification could reflect a difference in how they metabolize the drugs.

There are some limitations when interpreting the findings from our study. Depression diagnosis and antidepressant use was self-reported, which could lead to non-differential misclassification of the exposure. Even though we don’t have data on validity of self-reported antidepressant use in the Sister Study, a previous research study[49] compared the accuracy of self-reported with physician-reported antidepressant medication use and found substantial agreement between subject- and physician- reported antidepressant medication use (kappa = 0.60 ((95% (CI), 0.47–0.74); agreement = 80%). Thus, we expect reasonable accuracy of the antidepressant reports. Further, our use of reporting aids and the assembly of medications at time of the enrollment interview should have improved accuracy of reporting. Another limitation associated with AD use, is that we did not collect information on dosage, therefore, cannot assess dose-dependence. Type I error also is possible given the number of statistical tests performed.

A major strength of our study is the prospective design, with repeated ascertainment of antidepressant use and important confounders. Additionally, the large sample size, and large number of breast cancer cases allowed us to examine results separately by invasiveness, ER status, menopausal status, BMI and extent of first-degree family history of breast cancer. The use of the Sister Study also enhances power for detecting exposures that interact with genetic risk factors for breast cancer.

In conclusion, our data provides additional evidence that depression does not increase the risk of breast cancer. We found negative associations with postmenopausal breast cancer for antidepressant use, especially SSRIs. The apparent reduction in risk was greater among women with a stronger first-degree family history of breast cancer and among women with BMI < 25. Given the high prevalence of antidepressant use among postmenopausal women in the US, future studies need to confirm these associations and explore whether specific genetic pathways related to risk of breast cancer could be suppressed by SSRI.

## Supplementary Information

Supplementary Material 1. Table S1. Shows the results of the analyses assessing associations between history of clinical depression and breast cancer risk, after adjusting for use of antidepressants (Supplemental Table 1). Table S2 Show the results of the analyses evaluating the associations between antidepressant classes and breast cancer risk. It provides the Hazard Ratios and 95% CI, corresponding to the analyses showed in Figure 1. Table S3 Provides the results of the analyses evaluating the associations between AD use and breast cancer risk stratified by the lifetime duration of antidepressant use. Table S4 Provides the results of the analyses evaluating the associations between SSRI’s use and breast cancer risk stratified by the lifetime duration of antidepressant use.

## Data Availability

Collaborations based on data for the Sister Study are encouraged. The original dataset for the Sister Study is available from the National institute of Environmental Health Sciences. See https://sisterstudy.niehs.nih.gov/English/collaboration.htm.

## Abbreviations

SSRIs: Selective Serotonin Reuptake Inhibitors
TCAs: Tricyclic Antidepressants
BMI: Body Mass Index
CATI: Computer Assisted Telephone Interview
SNRIs: Serotonin and norepinephrine reuptake inhibitors
MET: Metabolic Equivalent of Task
AHEI: Alternative Healthy Eating Index
DCIS: Ductal Carcinoma In-Situ
ER: Estrogen Receptor
AD: Antidepressant
NHS: Nurses’ Health Study
