# Supplementary material for: Depression, antidepressant use, and breast cancer incidence in the Sister Study cohort
Source: Breast Cancer Res. 2025 May 15;27:82. doi: 10.1186/s13058-025-02043-4 (PMC12083028; doi:10.1186/s13058-025-02043-4)
Supplement: Supplementary file 1 — Supplementary Material 1. Table S1. Shows the results of the analyses assessing associations between history of clinical depression and breast cancer risk, after adjusting for use of antidepressants (Supplemental Table 1). Table S2 Show the results of the analyses evaluating the associations between antidepressant classes and breast cancer risk. It provides the Hazard Ratios and 95% CI, corresponding to the analyses showed in Figure 1. Table S3 Provides the results of the analyses evaluating the associations between AD use and breast cancer risk stratified by the lifetime duration of antidepressant use. Table S4 Provides the results of the analyses evaluating the associations between SSRI’s use and breast cancer risk stratified by the lifetime duration of antidepressant use. [file 13058_2025_2043_MOESM1_ESM.docx]

|  | **Clinical Depression** | |
| --- | --- | --- |
|  | **Exposed/Non-Exposed Cases** | **HR (95% CI)** |
| Overallᵃ | 917/2982 | 1.02 (0.94, 1.11) |
| DCIS | 190/630 | 1.05 (0.88, 1.26) |
| Invasive | 724/2347 | 1.01 (0.92, 1.11) |
| ER positiveᵇ | 549/1743 | 1.01 (0.91, 1.13) |
| ER negativeᵇ | 81/293 | 0.92 (0.68, 1.22) |
| Overall Premenopausal | 126/464 | 0.97 (0.78, 1.21) |
| Overall Postmenopausal | 791/2518 | 1.03 (0.94, 1.12) |
| Invasive Premenopausal | 96/362 | 0.94 (0.73, 1.22) |
| Invasive Postmenopausal | 628/1985 | 1.02 (0.92, 1.13) |

|  | | **No AD (ref)** | **SSRI Alone** | | | **SNRI Alone** | | | **TCA Alone** | | | **Miscellaneous Alone** | | | **Combinations** | | |
| --- | --- | --- | --- | --- | --- | --- | --- | --- | --- | --- | --- | --- | --- | --- | --- | --- | --- |
|  | | **Cases** | **Cases** | **HR (95%CI)** | **Het** | **Cases** | **HR (95%CI)** | **Het** | **Cases** | **HR (95%CI)** | **Het** | **Cases** | **HR (95%CI)** | **Het** | **Cases** | **HR (95%CI)** | **Het** |
| Overall^a^ | Overall | 3111 | 385 | 0.90 (0.81, 1.00) | . | 124 | 0.94 (0.78, 1.12) | . | 72 | 1.13 (0.90, 1.43) | . | 89 | 0.81 (0.65, 1.00) | . | 118 | 0.97 (0.81, 1.17) | . |
|  | BMI <25kg/m² | 1241 | 103 | 0.72 (0.59, 0.89) | . | 34 | 0.92 (0.66, 1.30) | . | 17 | 0.79 (0.49, 1.28) | . | 25 | 0.63 (0.43, 0.94) | . | 34 | 0.93 (0.66, 1.30) | . |
|  | BMI ≥25kg/m² | 1870 | 282 | 1.01 (0.89, 1.15) | 0.01 | 90 | 0.97 (0.78, 1.20) | 0.87 | 55 | 1.32 (1.01, 1.72) | 0.07 | 64 | 0.90 (0.70, 1.16) | 0.16 | 84 | 1.02 (0.82, 1.27) | 0.69 |
|  | 1 1st Degree Relative | 2009 | 260 | 0.98 (0.85, 1.11) | . | 71 | 0.87 (0.69, 1.10) | . | 44 | 1.15 (0.85, 1.54) | . | 58 | 0.81 (0.62, 1.05) | . | 80 | 1.04 (0.83, 1.30) | . |
|  | >1 1st Degree Relatives | 1102 | 125 | 0.78 (0.65, 0.94) | 0.07 | 53 | 1.04 (0.79, 1.37) | 0.31 | 28 | 1.09 (0.75, 1.58) | 0.82 | 31 | 0.80 (0.56, 1.14) | 0.98 | 38 | 0.85 (0.61, 1.18) | 0.37 |
| Invasive | Overall | 2443 | 311 | 0.92 (0.82, 1.04) | . | 101 | 0.97 (0.80, 1.19) | . | 55 | 1.09 (0.84, 1.42) | . | 68 | 0.78 (0.62, 1.00) | . | 93 | 0.97 (0.79, 1.19) | . |
|  | BMI <25kg/m² | 942 | 78 | 0.73 (0.57, 0.92) | . | 31 | 1.12 (0.78, 1.60) | . | 10 | 0.61(0.33, 1.13) | . | 21 | 0.70 (0.46, 1.08) | . | 24 | 0.86 (0.57, 1.30) | . |
|  | BMI ≥25kg/m² | 1501 | 233 | 1.03 (0.90, 1.19) | 0.01 | 70 | 0.94 (0.74, 1.20) | 0.41 | 45 | 1.34 (1.00, 1.80) | 0.02 | 47 | 0.82 (0.62, 1.10) | 0.57 | 69 | 1.04 (0.82, 1.33) | 0.45 |
|  | 1 1st Degree Relative | 1586 | 217 | 1.02 (0.89, 1.18) | . | 60 | 0.93 (0.72, 1.20) | . | 31 | 1.01 (0.71, 1.44) | . | 42 | 0.75 (0.55, 1.01) | . | 65 | 1.06 (0.82, 1.36) | . |
|  | >1 1st Degree Relatives | 857 | 94 | 0.75 (0.60, 0.93) | 0.02 | 41 | 1.03 (0.75, 1.42) | 0.56 | 24 | 1.20 (0.80, 1.79) | 0.54 | 26 | 0.85 (0.58, 1.26) | 0.53 | 28 | 0.80 (0.55, 1.18) | 0.27 |

|  | | **No AD time (ref)** | **<= 4 Years** | | **> 4 Years** | |  |
| --- | --- | --- | --- | --- | --- | --- | --- |
|  | | **Cases** | **Cases** | **HR (95%CI)** | **Cases** | **HR (95%CI)** | **Het.** |
| Overall^a^ | Overall | 2765 | 372 | 0.95 (0.85, 1.06) | 762 | 0.93 (0.86, 1.01) | 0.17 |
|  | BMI <25kg/m² | 1122 | 127 | 0.92 (0.77, 1.11) | 205 | 0.83 (0.71, 0.96) | 0.04 |
|  | BMI ≥25kg/m² | 1643 | 245 | 0.97 (0.84, 1.11) | 557 | 0.99 (0.89, 1.09) | 0.87 |
|  | 1 1st Degree Relative | 1795 | 240 | 0.95 (0.83, 1.09) | 487 | 0.93 (0.84, 1.03) | 0.35 |
|  | >1 1st Degree Relatives | 970 | 132 | 0.93 (0.77, 1.12) | 275 | 0.92 (0.80, 1.06) | 0.43 |
| Invasive | Overall | 2172 | 296 | 0.96 (0.85, 1.09) | 603 | 0.92 (0.84, 1.01) | 0.23 |
|  | BMI <25kg/m² | 851 | 97 | 0.93 (0.75, 1.15) | 158 | 0.83 (0.70, 0.99) | 0.11 |
|  | BMI ≥25kg/m² | 1321 | 199 | 0.98 (0.85, 1.14) | 445 | 0.98 (0.87, 1.09) | 0.90 |
|  | 1 1st Degree Relative | 1413 | 196 | 0.99 (0.85, 1.16) | 392 | 0.94 (0.84, 1.05) | 0.57 |
|  | >1 1st Degree Relatives | 759 | 100 | 0.90 (0.73, 1.11) | 211 | 0.89 (0.76, 1.04) | 0.27 |

|  | | **No time (ref)** | **<= 4 Years** | | **> 4 Years** | |  |
| --- | --- | --- | --- | --- | --- | --- | --- |
|  | | **Cases** | **Cases** | **HR (95%CI)** | **Cases** | **HR (95%CI)** | **Het.** |
| Overall^a^ | Overall | 3095 | 303 | 0.99 (0.88, 1.12) | 501 | 0.96 (0.87, 1.06) | 0.70 |
|  | BMI <25kg/m² | 1213 | 115 | 1.11 (0.91, 1.34) | 126 | 0.81 (0.67, 0.97) | 0.04 |
|  | BMI ≥25kg/m² | 1882 | 188 | 0.94 (0.81, 1.09) | 375 | 1.04 (0.93, 1.16) | 0.54 |
|  | 1 1st Degree Relative | 1993 | 202 | 1.05 (0.91, 1.22) | 327 | 0.98 (0.87, 1.11) | 0.75 |
|  | >1 1st Degree Relatives | 1102 | 101 | 0.89 (0.73, 1.10) | 174 | 0.92 (0.78, 1.08) | 0.36 |
| Invasive | Overall | 2441 | 236 | 0.98 (0.86, 1.12) | 394 | 0.95 (0.85, 1.05) | 0.58 |
|  | BMI <25kg/m² | 927 | 88 | 1.11 (0.89, 1.39) | 91 | 0.76 (0.61, 0.94) | 0.02 |
|  | BMI ≥25kg/m² | 1514 | 148 | 0.93 (0.78, 1.10) | 303 | 1.04 (0.91, 1.17) | 0.54 |
|  | 1 1st Degree Relative | 1574 | 162 | 1.07 (0.91, 1.26) | 265 | 1.00 (0.87, 1.14) | 0.71 |
|  | >1 1st Degree Relatives | 867 | 74 | 0.83 (0.65, 1.05) | 129 | 0.85 (0.71, 1.03) | 0.10 |
